# Non-photochemical quenching of photosystem I as an adaptive response to prolonged drought

**DOI:** 10.1093/jxb/erac438

**Published:** 2022-12-19

**Authors:** Juan B Arellano

**Affiliations:** Departamento de Estrés Abiótico, Instituto de Recursos Naturales y Agrobiología de Salamanca (IRNASA-CSIC), Cordel de Merinas 52, 37008 Salamanca, Spain

**Keywords:** Drought, integrating sphere-equipped spectrofluorometer, *Jatropha curcas*, light harvesting complex, non-photochemical quenching, photoprotection, photosystem, zeaxanthin

## Abstract

This article comments on:

**Sapeta H, Yokono M, Takabayashi A, Ueno Y, Cordeiro AM, Hara T, Tanaka A, Akimoto S, Oliveira MM, Tanaka R.** 2023. Reversible down-regulation of photosystems I and II leads to fast photosynthesis recovery after long-term drought in *Jatropha curcas*. Journal of Experimental Botany **74**, 336–351.

**Excess excitation energy (EEE) dissipation in photosystem II (PSII) is well established, while it is less studied and documented for PSI. Using *Jatropha curcas*, a semi-evergreen, tropical, non-edible oil-seed-bearing shrub, Sapeta *et al.* (2023) provide evidence for the significance of energy quenching in PSI under prolonged drought. *Jatropha curcas* receives considerable attention for sustainable (green) manufacturing of biodiesel and is recognized for its high tolerance to drought, capable of arresting growth while maintaining steady shoot water content and green leaves after several weeks of water withdrawal. In search for the molecular grounds for the drought tolerance of *J. curcas*, the study addresses the pigment and protein composition of PSI and PSII, and the thermal dissipation mechanisms of EEE absorbed by the light-harvesting complex (LHCI and LHCII) antennae of PSI and PSII. Their biochemical and spectroscopic measurements let them conclude that thermal energy dissipation by non-photochemical quenching (NPQ) indeed exists in PSI-LHCI and is essential for minimizing PSI inactivation and securing survival of *J. curcas* under prolonged drought stress. The photoprotection of PSI-LHCI by NPQ, together with the NPQ of PSII-LHCII and the prompt protein recovery of the PSII core complex, permit *J. curcas* plants to restart leaf CO**_**2**_ **assimilation and growth shortly after soil re-watering and stomatal re-opening.**

Plants are sessile organisms exposed to a plethora of environmental cues to which they flexibly respond in their seasonal life cycle. They either arrest growth when unfavorable growing conditions develop, or stimulate growth if conditions return to being favorable. NPQ of EEE as heat or chlorophyll fluorescence is a physiological photoprotection response of plants, also described in algae and cyanobacteria, through which the deleterious impact of EEE absorbed by LHC antennae on the photosynthetic apparatus is diminished (Demmig-Adams and Adams, 2006; Demmig-Adams *et al.*, 2014). At the molecular level, NPQ occurs in PSII-LHCII (Ruban, 2016; Demmig-Adams *et al.*, 2020). NPQ reduces the migration flow of EEE absorbed by LHCII antennae to the reaction center (RC) of PSII, hindering transient accumulation of singlet-excited chlorophyll in PSII-LHCII, which, in turn, could be converted to triplet-excited chlorophyll, and the subsequent PSII RC closure due to overaccumulation of reduced electron acceptor species in thylakoids.

Both triplet-excited chlorophyll and reduced electron acceptors can react with molecular oxygen to (photo)generate reactive oxygen species (ROS) (Arellano and Naqvi, 2016; Pospíšil *et al.*, 2022) that could cause photoinhibition of photosynthesis and deregulation of metabolism, and eventually lead to cell death if ROS are overproduced in an uncontrolled manner (Tyystjärvi, 2013; Mittler, 2017). NPQ consists of two main components, named qE and qI, with different functioning (Ruban, 2016; Ruban and Wilson, 2021). The flexible energy-dependent quenching, qE, is activated within seconds to a few minutes in PSII-LHCII under high light or stress conditions in which there is a decrease in ATP utilization. qE is triggered by conformational changes in thylakoid membranes after the protonation of the PsbS protein and the subsequent interaction of PsbS with major LHCII antennae, and by the accumulation of zeaxanthin in LHCII antennae after enzymatic de-epoxidation of violaxanthin to zeaxanthin, via antheraxanthin (Niyogi *et al.*, 1997; Li *et al.*, 2000). Both molecular processes depend on strong acidification of the thylakoid lumen and concomitant increase in the pH gradient (ΔpH) across the thylakoid membrane, in which cyclic electron transport around PSI was established to play a relevant role (Sato *et al.*, 2014). The slow inhibitory component of NPQ, qI, is irreversible and results from the photodamage to the PSII RC.

The overall time (of a few hundreds of picoseconds) for the excitation energy trapping in intact thylakoid-embedded PSII-LHCII complexes (Van Oort *et al.*, 2010) is nearly one order of magnitude longer than that (of a few tens of picoseconds) for PSI-LHCI (Jennings *et al.*, 2013). Indeed, PSI-LHCI is a relatively weak chlorophyll fluorescence emitter at ambient temperature. This implies that singlet-excited chlorophyll accumulates at lower levels in LHCI, and the probability for the build-up of triplet-excited chlorophyll through intersystem crossing is also lower. Therefore, the formation of ROS (i.e. singlet oxygen) should be less efficient in PSI-LHCI than in PSII-LHCII. In this context, elucidating whether or not PSI-LHCI can realize NPQ is the challenge faced by Sapeta *et al*. (2023) and other plant spectroscopists.

Time-resolved fluorescence measurements to follow NPQ in PSI-LHCI of *Arabidopsis thaliana* were already attempted, but different conclusions were drawn in two different studies (Ballottari *et al.*, 2014; Tian *et al.*, 2017). In the *npq2* mutant of *A. thaliana*, zeaxanthin was claimed to induce NPQ in LHCI when connected to PSI (Ballottari *et al.*, 2014), but no evidence was found for NPQ in PSI-LHCI of wild-type plants even when PSI-LHCI accumulated zeaxanthin molecules after the plants were exposed to a short high light stress (Tian *et al*., 2017). Because of the divergent conclusions on the biological significance of the potential role of zeaxanthin in NPQ in PSI-LHCI, Sapeta *et al*. (2023) endeavored to use an alternative spectroscopic approach with PSI-LHCI of *J. curcas*. This shrub can stand prolonged periods of drought by up-regulating abscisic acid (ABA) synthesis, chlorophyll metabolism, and ROS-detoxifying enzymes (Sapeta *et al.*, 2016). An important finding of particular interest for the spectroscopic analysis of NPQ in PSI-LHCI was that this photosynthetic complex in *J. curcas* could accumulate large amounts of zeaxanthin by efficient conversion of violaxanthin to zeaxanthin and *de novo* synthesis of zeaxanthin during exposure to water deficit for weeks (Sapeta *et al.*, 2023). This represents a notable advantage compared with the former two studies performed on *A. thaliana*, in which either zeaxanthin was constitutively present in *npq2* at high levels independent of the light treatment, or zeaxanthin accumulation in PSI-LHCI of wild-type *A. thaliana* plants briefly exposed to high light stress was lower than that found in PSI-LHCI of *J. curcas* after prolonged water withdrawal (~2–2.4 zeaxanthin molecules per 100 chlorophyll molecules).

Compelling evidence for NPQ in PSI-LHCI of *J. curcas* was obtained after monitoring the difference in fluorescence intensity at low temperature between purified PSI-LHCI complexes of control and drought-stressed plants using an integrating sphere with which the numbers of photons absorbed by both complexes were normalized. Another important finding was that zeaxanthin remained high during darkness in *J. curcas* (Sapeta *et al.*, 2023), suggesting that, together with flexible NPQ in PSII-LHCII, sustained, ΔpH-independent thermal dissipation also occurred in *J. curcas* during drought stress as observed in evergreen species adapted to endure unfavorable conditions over their seasonal life cycle (Demmig-Adams and Adams, 2006). Intriguingly, the kinetic analysis of the low temperature decay-associated spectra showed a significant decrease in the overall fluorescence lifetime of PSII-LHCII after prolonged drought stress, while the overall lifetime of PSI-LHCI showed insignificant variation, a result that Sapeta *et al*. (2023) rationalized by claiming that NPQ in PSI-LHCI occurred very rapidly and below the 5 ps resolution limit of their fluorescence analysis.

Sapeta *et al*. (2023) show that a large amount of zeaxanthin was still present in 11 h dark-adapted leaves and that flexible and sustained thermal dissipation mechanisms (Demmig-Adams and Adams, 2006) probably co-exist in *J. curcas*. Although no attempts were made to quantify zeaxanthin in isolated photosynthetic complexes (by large pore clear native PAGE) from 11 h dark-adapted leaves, this finding now opens the door to discuss whether or not the loss in zeaxanthin accumulation during dark incubation was uniform between both PSI-LHCI and PSII-LHCII complexes. Both flexible and sustained thermal dissipation mechanisms could (equally) occur in PSI-LHCI and PSII-LHCII complexes. However, no evidence for flexible thermal dissipation was observed in PSI-LHCI of wild-type *A. thaliana* plants characterized by their short life cycle (Tian *et al.*, 2017). This also raises the questions of whether NPQ in PSI-LHCI is mainly present in (semi-)evergreen species, such as *J. curcas*, exposed to unfavorable seasonal/growing conditions or whether the NPQ in PSI-LHCI is sustained and ΔpH independent because of the intensity and duration of the stress. The absence of variation in zeaxanthin accumulation in PSI-LHCI during prolonged stress periods could avoid severe PSI photoinactivation and reduce the energy cost of protein biosynthesis.

Ultrafast NPQ in PSI-LHCI (<10 ps) was claimed to occur in *J. curcas* in order to rationalize the decrease in the fluorescence rise amplitude at ~738 nm (Sapeta *et al.*, 2023). Future lines of research should aim to experimentally confirm by femtosecond time-resolved spectroscopy that the zeaxanthin accumulation in PSI-LHCI is in fact responsible for the fluorescence quenching of the red-most emitting chlorophyll molecules and that its time constant is even shorter than the 12 ps described in the *npq2* mutant of *A. thaliana* (Ballottari *et al.*, 2014). While in *npq2* and wild-type *A. thaliana* briefly exposed to high light stress, zeaxanthin was present constitutively in LHCI (Ballottari *et al*., 2014) or was partly displacing violaxanthin molecules from their specific binding sites in LHCI (Tian *et al.*, 2017), Sapeta *et al*. (2023) indicate that, together with the conversion of pre-existing violaxanthin to zeaxanthin, there was *de novo* synthesis of zeaxanthin in *J. curcas* under drought stress. The stoichiometric zeaxanthin:PSI-LHCI ratio was variable and ranged between ~2.0 and 2.4 zeaxanthin molecules per 100 chlorophylls in *J. curcas*, and this could indicate that there were zeaxanthin molecules occupying extra binding pockets at the protein–protein interface between LHCI and PSI. Some hypotheses on the location and mode of action of zeaxanthin in NPQ in PSII-LHCII were put forward by Xu *et al.* (2015), in which zeaxanthin was proposed to enhance aggregation and act directly as a quencher in thylakoid membranes. If zeaxanthin performs better in NPQ in LHCI while binding at the interface between LHCI and PSI, this could support the ultra-fast NPQ in PSI-LHCI of *J. curcas* and probably PSI-LHCI conformational changes under prolonged drought stress. The presence of extra binding pockets for zeaxanthin in PSI-LHCI and the stoichiometric variability necessitate performing circular dichroism and femtosecond time-resolved analyses with leaves or photosynthetic preparations of (semi-)evergreen species in the future in which PSI-LHCI should remain unperturbed by detergent treatments.

The presence of NPQ beyond PSII-LHCII in *J. curcas* under drought stress opens the door to explore whether NPQ in PSI-LHCI is a more general photoprotection mechanism found in (semi-)evergreen species exposed to prolonged stress conditions.
